# Nasal Mucosal Heavy Metal Accumulation, Olfactory Dysfunction, and Histopathological Changes: A Prospective Clinical Study

**DOI:** 10.3390/jcm15145457

**Published:** 2026-07-12

**Authors:** Goran Malvić, Svjetlana Janković, Damir Klepac, Dijana Tomić Linšak, Marin Tota, Željko Linšak, Blažen Marijić, Dubravko Manestar

**Affiliations:** 1 Clinical Hospital Centre Rijeka, Clinic of Otorhinolaryngology and Head and Neck Surgery, 51000 Rijeka, Croatia; goran.malvic@medri.uniri.hr (G.M.); blazen.marijic@uniri.hr (B.M.); dubravko.manestar@medri.uniri.hr (D.M.); 2 Faculty of Medicine, University of Rijeka, Braće Branchetta 20, 51000 Rijeka, Croatia; damir.klepac@uniri.hr (D.K.); dijanatl@uniri.hr (D.T.L.); marin.tota@uniri.hr (M.T.); zeljko.linsak@uniri.hr (Ž.L.); 3 Centre for Micro- and Nanosciences and Technologies, University of Rijeka, Radmile Matejčić 2, 51000 Rijeka, Croatia

**Keywords:** heavy metals, environmental exposure, olfactory dysfunction, nasal mucosa, rhinomanometry

## Abstract

**Background/Objectives**: Heavy metals are environmental hazards that are recognized as neurotoxic agents that may impair olfactory pathways in susceptible individuals. This study aimed to determine the concentrations of cadmium (Cd), arsenic (As), lead (Pb), chromium (Cr), manganese (Mn), nickel (Ni), copper (Cu), zinc (Zn), iron (Fe), and mercury (Hg) in the nasal mucosa and secretions of patients diagnosed with nasal septal deviation, nasal polyposis, or chronic rhinosinusitis, and to investigate their associations with olfactory function, nasal airflow, and pathohistological changes in the nasal mucosa. **Methods**: This prospective study included 97 adult patients who underwent nasal surgery. We used the Smell Diskettes Olfaction Test to assess olfactory function and rhinomanometry to evaluate nasal airflow resistance. Nasal mucosal tissue samples were collected intraoperatively. Heavy metal concentrations were analyzed using inductively coupled plasma mass spectrometry and atomic absorption spectrometry. **Results**: Preoperative olfactometry scores in patients with olfactory dysfunction differed to those in patients without olfactory dysfunction (3.4 ± 1.3 vs. 4.1 ± 1.2). Inferential analysis of heavy metals and olfactometry revealed that Cr, Fe, and Pb concentrations were negatively associated with olfactory performance before exposure (Cr: β = −0.12, *p* = 0.018; Fe: β = −0.08, *p* = 0.009; Pb: β = −0.10, *p* = 0.048). Rhinomanometry parameters showed positive associations with olfactory function, independently of age, sex, and groups of patients with or without olfactory dysfunction (R1: β = 0.45, *p* < 0.001; R2: β = 0.30, *p* = 0.003). Stepwise regression identified Fe, Pb, and Cr as the strongest predictors of olfactory dysfunction. The adverse effects of Fe and Pb exposure were significantly amplified with age. MANOVA revealed significant differences in heavy metal concentrations across pathohistological categories (Wilks’ Lambda = 0.038, *p* < 0.001). Carcinoma tissue exhibited significantly higher Cr concentrations, whereas precancerous lesions exhibited increased Cd concentrations. **Conclusions:** Cr, Fe, and Pb accumulation in nasal mucosa secretion is significantly associated with impaired olfactory function and pathohistological changes. Prolonged exposure to environmental heavy metals may affect olfactory function in the elderly population.

## 1. Introduction

The nasal cavities are highly innervated by adrenergic, cholinergic, and sensory neurons, which are responsible for protective reflexes. Air in the olfactory zone stimulates the neuro-olfactory epithelium and perception of smell. Smell is well known to play a protective role in the environment by enabling the detection of potentially harmful environmental exposures (toxic gases) [1]. The olfactory bulb is connected to the limbic system, hypothalamus, and autonomic nervous system; therefore, the sensory function contributes to behavior, emotion, nutrition, and safety. Moreover, the sense of smell is closely associated with psychosocial well-being and quality of life [1,2].

There is an increasing awareness of the prevalence of olfactory dysfunction as a clinical and public health problem due to global demographic shifts in aging populations [3,4,5]. Due to the complex neuroepithelial connections, olfactory dysfunction contributes to depression and social isolation in the aging population and may be an early clinical symptom of neurodegenerative disorders, such as Parkinson’s disease [2,6,7]. Recent studies have emphasized the importance of olfactory dysfunction in otorhinolaryngology and translational neuroscience [8,9,10].

The traditional classification of olfactory disorders includes conductive and sensorineural disorders [1,8]. Conductive olfactory dysfunction is caused by mucosal edema, chronic rhinosinusitis, nasal polyposis, or structural nasal abnormalities, causing irregular access of odorants to the olfactory cleft [11]. Sensorineural dysfunction arises from injury to the olfactory neuroepithelium, olfactory neurons, or central neuronal olfactory pathways [8,9]. The olfactory neuroepithelium, due to its specific anatomical localization connecting the external environment and central nervous system, is highly vulnerable to inhaled toxic agents and environmental pollutants [12,13,14].

Heavy metals are a group of environmental risk factors for human health and biological systems because they have persistent bioaccumulative potential and chronic toxic effects [15,16]. Humans are exposed to heavy metal toxicity not only through inhalation but also through ingestion; chronic tobacco smoke exposure; consumption of contaminated water; and, for workers in industrial sectors, various types of occupational settings. Regarding cadmium (Cd), chromium (Cr), and nickel (Ni) in the context of occupational exposure, there is strong evidence of their toxic effects causing olfactory mucosa damage and sinonasal pathological alterations [17], as well as evidence of arsenic (As), lead (Pb), manganese (Mn), copper (Cu), zinc (Zn), iron (Fe), and mercury (Hg) having neurotoxic, mutagenic, and carcinogenic properties [15,16,18]. Earlier studies were mostly concerned with the systemic toxicity and lower respiratory tract effects of heavy metals, while recent studies are more concerned with airborne pollutants, which may substantially and chronically affect the upper respiratory tract and olfactory system, causing chronic rhinosinusitis [19].

Emerging occupational and environmental studies have demonstrated impaired olfactory function in industrial workers with greater exposure not only to Cd, Ni, and Cr [17] but also to Mn, Hg, and As [17,20,21,22,23,24]. In industrial environments, exposure to Cd and Ni, due to their toxic effects on the nasal mucosa, was associated with anosmia and chronic inflammatory changes [17,20,21,22]. Studies of Mn toxicity in populations with chronic environmental and occupational exposure detected both peripheral dysfunction in odor detection and central olfactory dysfunction in odor identification in particular populations [23,24]. Chronic rhinitis or rhinosinusitis, different mucosal epithelial ulcerations, and septal perforations may be caused by corrosive industrial compounds inhaled during chronic occupational exposure [12,25,26]. Occupational and environmental studies have explored exposure through patient history or systemic biomarker levels in occupational cohorts; however, there are only limited quantitative studies on heavy metal accumulation within sinonasal mucosal tissue.

There are experimental studies supporting the biological probability of olfactory injury induced by heavy metals. Some animal and in vitro studies have demonstrated that inhaled nanoparticles and metal-containing aerosols may be transported through olfactory neuronal pathways directly to the brain, bypassing the blood–brain barrier and accumulating within the olfactory bulb and central nervous system [13,14]. Some studies have explored chronic inhalational exposure in association with histopathological alterations of the nasal mucosa, including squamous metaplasia, epithelial dysplasia, ulceration, and precancerous lesions [27,28]. Along with their contribution to olfactory dysfunction, these studies demonstrate that heavy metal accumulation acts as a stressor to the olfactory epithelium, causing its replacement with a more protective respiratory epithelium and causing further pathological changes [28]. These clinical studies also demonstrated higher trace element concentrations in chronic rhinosinusitis with or without nasal polyps [29,30,31].

Despite the broad range of occupational and environmental studies, there is a gap in comprehensive studies integrating quantitative heavy metal analysis in nasal mucosal tissue, objective olfactory testing, rhinomanometry, and histopathological evaluation. There are no standardized clinical approaches for evaluating and monitoring patients with olfactory dysfunction that may be associated with environmental exposure [8,10,32,33,34].

The present prospective study aims to quantify the concentrations of Cd, As, Pb, Cr, Mn, Ni, Cu, Zn, Fe, and Hg in nasal mucosa samples obtained from patients undergoing nasal surgery. One of the main aims of this study was to investigate the relationship between tissue heavy metal accumulation and olfactory function using olfactometry assessment [32]. The specific aim of this study was to explore the association between heavy metal concentrations, nasal airflow parameters, and histopathological changes in the nasal mucosa. The hypothesis of this study is that increased heavy metal accumulation within the nasal mucosa is associated with olfactory dysfunction and pathological changes in the nasal mucosal tissue.

## 2. Materials and Methods

### 2.1. Study Design and Patients

This prospective observational study was conducted at the Clinic of Otorhinolaryngology and Head and Neck Surgery (Clinic), Clinical Hospital Center Rijeka (CHC), in collaboration with the Teaching Institute of Public Health (TIPH) of Primorje-Gorski Kotar County. The study procedures were conducted in accordance with the ethical standards of the institutional and national research committees and the Declaration of Helsinki. The study protocol was approved by the Institutional Ethics Committee of the Clinical Hospital Centre Rijeka, Croatia (approval number: 2170-29-02/1-23-2), and the Ethics Committee of the Teaching Institute of Public Health of Primorje-Gorski Kotar County (approval number: 08-880-139/4-23). Written informed consent was obtained from all patients prior to participation in the study.

During the study period, 97 adult patients diagnosed with nasal septal deviation, nasal polyposis, or chronic rhinosinusitis were selected. All patients underwent nasal surgery, functional endoscopic sinus surgery (FESS), or septoplasty, depending on the underlying pathology. All patients underwent a standardized preoperative clinical evaluation prior to surgical treatment. On the day of hospital admission, before surgery, the baseline clinical examinations were repeated.

Nasal mucosa samples were collected by surgeons intraoperatively under sterile conditions. In patients with chronic rhinosinusitis (CRS) with nasal polyps (CRSwNP) or without nasal polyps (CRSsNP), tissue samples were collected from the affected mucosa, mainly from the middle nasal concha area. In patients diagnosed with septal deviation, healthy respiratory epithelium was collected from the nasal septum during septoplasty, and these samples served as the control samples.

Nasal mucosa samples from all 97 patients were separated for either elemental laboratory analyses at the Teaching Institute of Public Health (TIPH) or histopathological analyses at the Clinical Institute of Pathology and Cytology, CHC Rijeka. At the TIPH, quantitative elemental analyses of heavy metal concentrations in nasal mucosa samples were performed using inductively coupled plasma mass spectrometry (ICP-MS). Trace element quantification was performed according to standardized digestion and mineralization procedures, and the concentrations of the examined heavy metals were expressed in standardized units relative to tissue mass.

Histopathological analyses were conducted at the Clinical Institute of Pathology and Cytology after standard processing methods, which included fixation of tissues in formalin, sectioning, and staining with hematoxylin and eosin. Microscopic analyses of tissue samples were performed, and according to the degree of pathological alternation, they were categorized as metaplastic, dysplastic, premalignant (precancerous), or malignant (cancerous) lesions.

For patient selection, the exclusion criteria included age < 18 years, previous nasal surgery or oncological treatment (radiotherapy or chemotherapy) in the head and neck region, and current malignant disease. This stratification enabled a comparative assessment of clinical parameters; olfactory function; nasal airflow; histopathological findings; and demographic parameters, including age, sex, and occupational exposure status.

### 2.2. Preoperative Clinical Assessment of Patients

All patients included in this study underwent preoperative olfactometry, rhinomanometry, and nasal endoscopy diagnostic evaluation.

Olfactory function was assessed twice for each patient: first during the preoperative diagnostic examination and again on the day of hospital admission immediately before surgery. First, we performed a standardized olfactometry assessment test, the Smell Diskettes Olfaction Test (SDOT, Novimed, Dietikon, Switzerland), which includes eight odorants (coffee, vanilla, peach, grass, pineapple, rose, chocolate, and fish) accompanied by a structured questionnaire. The concentration of the odor in the tile of the SDOT was significantly higher than the odor threshold itself. Each correct answer was awarded one point, and the maximum number of points was eight. A score of 7 and 8 points represents normal olfactory function, while a score of 6 points or less is indicative of hyposmia or anosmia.

As the second diagnostic method, we performed rhinomanometry to obtain accurate values of nasal breathing quality and to objectively assess nasal obstruction in each patient. This was performed twice for each patient: during the outpatient preoperative diagnostic examination (R1 value) and again on the day of hospital admission before surgery (R2 value). It was performed using a Rhinomanometer 300 (Atmos, Lenz Kirch, Germany) to assess the impact of nasal cavity obstruction on olfactory dysfunction by evaluating nasal airflow, pressure, and resistance. Using the values obtained with rhinomanometry, our goal was to compare the impact of respiratory obstruction with increased concentrations of heavy metals on the nasal mucosa and olfactory dysfunction.

Nasal endoscopy, as the third diagnostic method, was performed with the aim of better visualizing less accessible places in the nose, such as the posterior regions of the nasal cavum and the olfactory region of the nose, and was used to classify septal deviation and determine the degree of nasal polyposis. The procedure was performed under topical epimucosal anesthesia.

On the day of hospital admission, before surgery, demographic and occupational data of all patients, including age, sex, smoking status, occupation, and place of residence, were collected to evaluate the potential heavy metal exposure of each patient. Repeated baseline examinations on the day of hospital admission were performed as part of standardized baseline measurements immediately prior to tissue sampling and surgery.

### 2.3. Surgical Procedure and Laboratory Analysis

At the beginning of the surgical procedure, a sample of the nasal mucosa, along with naturally accumulated secretions, which are routinely removed to improve airway patency of patients, were collected either from the middle nasal concha or from the nasal septum. The materials from these anatomical sites were selected because of their direct exposure to inhaled air, making them representative for the assessment of heavy metal accumulation.

Tissue samples taken during the surgical procedure were stored in plastic containers and transported to the laboratory. In the laboratory, the samples were immediately frozen in liquid nitrogen and stored at −80 °C. Before analysis for heavy metal concentrations, the samples were disrupted using an Anton Paar Multiwave 3000 microwave system (Perkin Elmer, Shelton, CT, USA) equipped with pressure vessels, using 5 mL of 65% nitric acid per sample (HNO3 Suprapur, Merck, Darmstadt, Germany), for 20 min in a 200 °C operating cycle. The disrupted samples were transferred to a 25 mL volumetric flask containing ultrapure water (Siemens).

In this study, the heavy metals Cd, As, Pb, Cr, Mn, Ni, Cu, Zn, and Fe were analyzed due to their already established toxicity, which may potentially contribute to inflammatory and allergic diseases, as well as to olfactory dysfunction. Their concentrations in the tissue samples were determined using an inductively coupled plasma mass spectrometer (ICP-MS; NexION 2000X, Perkin Elmer, USA) equipped with an S10 autosampler (Perkin Elmer, USA).

To determine mercury (Hg) content, 0.1 g of lyophilized tissue was weighed and transferred directly into an analyzer vessel and analyzed using an AMA 254 atomic absorption spectrometer (Advanced Mercury Analyzer, Leco, St. Joseph, MI, USA).

The analyses enabled the comparison of heavy metal concentrations according to sex, age group, occupational exposure, and patient cohort, as well as the assessment of their association with olfactory function and nasal airflow parameters.

### 2.4. Statistical Analysis

A detailed comparative assessment of the clinical and demographic parameters was performed. Data collection and entry were performed using Microsoft Excel prior to the statistical analysis. Statistical analyses were conducted using Data Science Workbench software (version 14, Cloud Software Group, Inc., Fort Lauderdale, FL, USA, 2025). Statistical significance was set at *p* < 0.05. For the heavy metal distribution across pathohistological findings, we used MANOVA, ANOVA, and Tukey HSD post hoc testing. For the analysis of predictors of olfactory dysfunction, we performed multivariable regression and inferential analysis for heavy metal concentrations/rhinomanometry/olfactometry and correlation analyses for heavy metal concentrations with rhinomanometry/olfactometry results.

## 3. Results

### 3.1. Study Population and Olfactometry/Rhinomanometry Findings

This study included 97 adult patients undergoing nasal surgery at the Clinic for Otorhinolaryngology and Head and Neck Surgery. A total of 42 patients with isolated nasal septal deviation served as the non-inflammatory control group, whereas 55 patients were diagnosed with chronic rhinosinusitis, namely, 25 with CRSsNP and 30 with CRSwNP. Based on preoperative olfactory testing, it was found that all patients with chronic rhinosinusitis exhibited olfactory dysfunction, whereas patients with isolated septal deviation had preserved olfactory function and therefore served as the control group.

Of the 97 patients, 58 (59.8%) were men and 39 (40.2%) were women. The patients were stratified into three age groups: 18–37 (*n* = 32), 38–55 (*n* = 33), and 56–78 years (*n* = 32). For each patient in both groups and across sex and age categories, olfactory and rhinomanometry measurements were performed before (OL_BE) and after (OL_AFT) testing. Baseline demographic characteristics, diagnosis distribution, olfactometry, and rhinomanometry findings are summarized in Table 1.

Preoperative olfactometry scores (OL_BE) were lower in patients with olfactory dysfunction than in those without (3.4 ± 1.3 vs. 4.1 ± 1.2). This finding confirmed the already diagnosed impaired olfactory function among affected patients. Preoperative olfactometry scores (OL_BE) were slightly higher in females than in males (3.9 ± 1.3. vs. 3.7 ± 1.2). Preoperative olfactory function differed according to age group. The youngest patient group (18–37 years) achieved the highest mean OL_BE scores (4.1 ± 1.1), while the oldest patient group (56–78 years) exhibited the lowest OL_BE scores (3.2 ± 1.4).

Post-test olfactometry scores (OL_AFT) were higher across all categories, but the trend was the same as that of the preoperative olfactometry scores (OL_BE). Patients with olfactory dysfunction had lower scores than those without (4.6 ± 1.3 vs. 5.1 ± 1.5). Female patients also demonstrated slightly higher post-test olfactometry scores (OL_AFT) than male patients (5.0 ± 1.5 vs. 4.9 ± 1.4). The youngest group had the highest OL_AFT values (5.2 ± 1.3), while the oldest group had the lowest scores (4.7 ± 1.5).

Rhinomanometry measurements did not show significant differences between the groups of patients or across the sex and age variables. The mean R1 and R2 values of both groups of patients revealed negligible differences in nasal airflow resistance (R1:0.52 ± 0.10 vs. 0.53 ± 0.11; R2:0.38 ± 0.10 vs. 0.40 ± 0.11).

The proportion of patients with olfactory dysfunction was highest among those with inflammatory diseases (CRSwNP and CRSsNP) and lowest among those with septal deviation.

### 3.2. Heavy Metal Concentrations in Samples of Nasal Mucosa

ICP-MS analysis was performed on the concentrations of the heavy metals Cd, As, Pb, Cr, Mn, Ni, Cu, Zn, and Fe in intraoperatively taken nasal mucosa samples of 97 patients (Table 2).

Among all heavy metal concentrations, the Cr, Mn, Fe, Ni, Cu, and Zn concentrations in patients with olfactory dysfunction were slightly higher than those in patients without olfactory dysfunction. For example, the mean Fe concentrations in patients with olfactory dysfunction differed from those in patients without olfactory dysfunction (111.0 ± 107.0 μg/g vs. 106.0 ± 103.0 μg/g), the mean Cr concentrations were elevated in patients with olfactory dysfunction compared to those in patients without olfactory dysfunction (0.24 ± 0.31 μg/g vs. 0.21 ± 0.29 μg/g), and the mean Ni concentrations were also higher in patients with olfactory dysfunction than in patients without olfactory dysfunction (0.74 ± 2.20 μg/g vs. 0.69 ± 1.95 μg/g). Higher heavy metal concentrations were detected in patients already diagnosed with impaired olfactory function.

Among all heavy metal concentrations, higher mean concentrations of Cr, Mn, Fe, Ni, and Pb were observed in male patients—for example, the mean concentrations of Cr were 0.22 ± 0.30 μg/g vs. 0.20 ± 0.28 μg/g, those of Fe were 110.0 ± 105.0 μg/g vs. 108.0 ± 100.0 μg/g, and those of Ni were 0.76 ± 2.25 μg/g vs. 0.68 ± 1.98 μg/g. In females, ICP-MS analysis revealed higher mean concentrations of Cu and Zn than in males.

Regarding age groups, differences in heavy metal accumulation were not pronounced throughout the study population. For example, Cr concentrations were lowest in the youngest age group compared to the oldest group of patients (0.18 ± 0.25 μg/g vs. 0.25 ± 0.33 μg/g), and similar patterns were detected for Mn, Ni, and Zn. Descriptive statistics for all heavy metals are presented in Table 2.

Data are presented as the mean ± standard deviation (SD). Complete data for all the analyzed heavy metal concentrations are presented in Table A1 (Appendix A).

### 3.3. Heavy Metal Distribution According to Clinical Diagnosis and Pathohistological Findings

A multivariate analysis of variance test (MANOVA) was performed to investigate whether tissues with different pathohistological categories showed different overall heavy metal profiles. The independent variables were pathohistological categories (precancerous lesions, metaplasia, dysplasia, carcinoma, and healthy respiratory mucosa as control samples taken from the mucosa of the reticuloendothelial system), and the dependent variables were heavy metal concentrations.

The MANOVA revealed that the combined concentrations of the analyzed metals differed significantly across the pathohistological categories. The results indicated that heavy metal accumulation in nasal mucosa samples was strongly associated with the pathohistological category (Wilks’ Lambda = 0.038, F(36, 316.52) = 12.177, *p* < 0.001). A univariate ANOVA was performed for individual metals and revealed significant differences between the PH categories (*p* < 0.05). Descriptive heavy metal concentrations according to pathohistological findings are presented in Table 3.

The highest Cr concentrations were observed in carcinoma samples (3.02 ± 2.08 μg/g), whereas Fe, Cu, and Zn were highest in healthy respiratory mucosa. Precancerous lesions showed a marked increase in Cd concentration (17.03 ± 37.94 μg/g), substantially exceeding all other tissue categories. In contrast, Mn, As, Ni, and Pb showed no statistically significant overall differences among pathohistological groups.

To identify which tissue categories differed significantly, Tukey’s HSD post hoc analysis was performed.

Significant pairwise comparisons are summarized in Table 4.

Post hoc analysis showed different heavy metal concentrations (Cr, Fe, Cd, Cu, and Zn) across pathohistological (PH) categories and in the healthy respiratory mucosa (all *p* < 0.001). Carcinoma tissue (*n* = 2) showed elevated Cr concentrations compared with metaplasia tissue (*n* = 26), dysplasia tissue (*n* = 22), precancerous tissue (*n* = 5), and healthy respiratory epithelium (*n* = 42), whereas the largest mean difference was observed between carcinoma and precancerous tissues (2.956 μg/g; 3.020 vs. 0.064 μg/g, *p* = 0.0001) and between carcinoma and healthy respiratory mucosa tissues (3.020 vs. 0.368 μg/g, *p* = 0.0001).

Healthy respiratory mucosa (*n* = 42) showed higher Fe, Cu, and Zn concentrations than dysplasia, metaplasia, and precancerous lesions. The Fe concentration in healthy respiratory mucosa tissue was 278.42 μg/g, while dysplasia samples exhibited 120.48 μg/g (*p* = 0.0016), metaplasia samples exhibited 86.43 μg/g (*p* = 0.0001), and precancerous lesion samples exhibited 39.06 μg/g (*p* = 0.0004).

Precancerous tissues (*n* = 5) showed the highest Cd concentrations compared with dysplastic (*p* = 0.0017), healthy respiratory mucosa (*p* = 0.0146), and metaplastic tissues (*p* = 0.0011). The Cd concentration in this group was 17.027 μg/g, whereas all other groups exhibited concentrations below 0.11 μg/g.

Pathohistological findings were interpreted in relation to the preoperative clinical diagnosis. Healthy respiratory mucosa was predominantly identified in patients undergoing surgery for septal deviation, metaplastic changes were most frequent in CRSsNP, and advanced epithelial lesions (high-grade dysplasia, precancerous lesions, and carcinoma) occurred predominantly in CRSwNP. This clinicopathological correlation enabled a comparison of characteristic heavy metal profiles across the three clinical diagnosis groups (Table 5). Patients with septal deviation (healthy respiratory mucosa phenotype) showed the highest baseline levels of Fe (278.4 ± 132.6 μg/g) and Cu (2.30 ± 1.01 μg/g), reflecting a physiological mucosal metal content without pronounced epithelial remodeling. Chromium and zinc levels were relatively low to moderate, while cadmium and lead levels remained minimal (Cd: 0.104 ± 0.062 μg/g; Pb: 0.11 ± 0.15 μg/g). In contrast, CRSsNP-associated tissue patterns (predominantly metaplasia with early dysplasia) were characterized by a marked reduction in Fe (86.4 ± 59.5 μg/g) and Cu (0.87 ± 0.42 μg/g), alongside lower Zn concentrations (3.87 ± 2.24 μg/g). These findings suggest a depletion of essential trace elements in chronic inflammatory remodeling without advanced epithelial transformation.

The CRSwNP group (dysplasia, precancerous lesions, and carcinoma-associated profiles) demonstrated the most pronounced heavy metal dysregulation. Carcinoma-associated samples exhibited a strong accumulation of Cr (3.02 ± 2.08 μg/g) and Ni (17.73 ± 9.84 μg/g), indicating potential metal-related genotoxic stress. Precancerous lesions showed an extreme elevation in Cd (17.03 ± 37.94 μg/g), significantly exceeding all other groups, suggesting cadmium accumulation as a possible marker of early malignant transformation. Zn levels were moderately increased in CRSwNP-related tissues (5.36 ± 2.23 μg/g), consistent with tissue repair and inflammatory activation.

Overall, healthy respiratory mucosa corresponding to septal deviation exhibited the highest concentrations of Fe, Cu, and Zn, representing the physiological metal profile of non-remodeled nasal mucosa. In contrast, CRSsNP demonstrated depletion of essential trace elements, particularly Fe and Cu, whereas CRSwNP was characterized by progressive accumulation of Cr, Ni, and Cd, indicating increasing oxidative stress and possible metal-related epithelial transformation. Among all analyzed metals, cadmium demonstrated the most pronounced increase, with a marked elevation in precancerous lesions. Pb and As showed no consistent distribution across the estimated clinical etiologies.

### 3.4. Heavy Metals, Rhinomanometry Parameters, and Olfactory Function Associations

Inferential analyses were performed to evaluate the associations between heavy metal concentrations in the nasal mucosa, rhinomanometry parameters, demographic variables, and preoperative olfactory performance (OL_BE). Higher concentrations of Cr, Fe, and Pb in nasal mucosa samples from 97 patients were significantly associated with lower olfactory scores. In addition, older age and the presence of previously diagnosed olfactory dysfunction were associated with reduced olfactory performance, whereas sex was not a significant predictor. Table 6 shows that negative associations with OL_BE scores were observed for Cr (β = −0.12, *p* = 0.018), Fe (β = −0.08, *p* = 0.009), and Pb (β = −0.10, *p* = 0.048). Analyses revealed that older patients had reduced olfactory performance (β = −0.15, *p* = 0.014). Significantly lower OL_BE scores (β = −0.20, *p* = 0.005) were observed in patients with already diagnosed olfactory dysfunction (*n* = 55) than in patients without already diagnosed olfactory dysfunction (*n* = 42). The sex variable was not significantly associated with olfactory outcomes in either group.

Inferential analyses examining rhinomanometry and olfactometry parameters showed significant positive associations with OL_BE scores regarding age and sex variables in both groups of patients: higher R1 values were associated with better olfactory function (β = 0.45, *p* < 0.001), and R2 values were also positively associated with OL_BE scores (β = 0.30, *p* = 0.003). Age was negatively associated with olfactory outcomes in both groups.

Stepwise regression analysis was performed to determine which heavy metals were predominantly associated with olfactory dysfunction. The results revealed that the tested Fe concentrations were the strongest predictors of olfactory dysfunction. Fe accounted for 14.5% of the variance in OL_BE scores in the initial model (R^2^ = 0.145, *p* < 0.001).

Interaction analyses revealed significant Fe × age (β = −0.06, *p* = 0.048) and Pb × age (β = −0.08, *p* = 0.048) interactions, indicating stronger negative associations between these metals and olfactory performance in older patients than in younger patients. No statistically significant interactions were observed regarding sex or previously diagnosed olfactory dysfunction.

Correlation analyses revealed slightly positive correlations among Cr, Mn, and Ni, which may indicate a common environmental or occupational exposure in these patients. Significant negative correlations were observed between heavy metal concentrations and OL_BE scores, whereas rhinomanometry parameters showed positive correlations with olfactory performance. The correlations between rhinomanometry parameters and heavy metal concentrations were comparatively weaker. The complete correlation matrix is presented in Table A2.

## 4. Discussion

This prospective study demonstrated significant associations between heavy metal accumulation within nasal mucosal tissue and olfactory function in patients undergoing surgery for chronic rhinosinusitis or septal deviation. The results showed that higher concentrations of Cr, Fe, and Pb were independently associated with lower preoperative olfactory scores (OL_BE) and that older age further contributed to reduced olfactory performance in these patients.

Statistical results from stepwise regression identified Fe as the strongest predictor of olfactory dysfunction, as it independently accounted for 14.5% of the variation in olfactory scores. Multivariable statistical analysis including Fe, Pb, Cr, and age explained approximately one-third of the total variance in olfactory performance in 97 patients (R^2^ = 0.315, *p* < 0.001). These results support the hypothesis that prolonged exposure to neurotoxic environmental heavy metals may substantially contribute to olfactory impairment [17,20,35]. Although the explained variance was moderate (R^2^ = 0.315), these findings indicate that heavy metal exposure represents one of several factors contributing to olfactory dysfunction.

Along with the clinical relevance of these findings, olfactory dysfunction is increasingly recognized as an important public health problem associated with impaired quality of life, and nutritional and psychosocial problems [2,6,7,35]. Moreover, environmental toxic exposure may contribute to neuroepithelial damage and progressive olfactory dysfunction and may be linked to early neurodegenerative disorders [12,13,14].

The relationship between heavy metal accumulation and olfactory function is strengthened by age-dependent interactions with heavy metal concentrations. In particular, among older patients, the findings revealed that significant Fe × age and Pb × age interactions were associated with more pronounced negative effects of heavy metal accumulation on olfactory performance, suggesting that aging processes may increase susceptibility to heavy metal toxicity. Physiological changes associated with aging include progressive degeneration of olfactory neurons, compromised regenerative capacity of the olfactory epithelium, and cumulative chronic exposure to various environmental toxins [3,5,35]. These findings may raise awareness of the effects of environmental heavy metal exposure in elderly populations and provide implications for public health preventive measures.

Inferential statistical analyses revealed that sex was not a statistically significant predictor of olfactory performance, although female patients exhibited slightly higher olfactometry scores than male patients. Variations in heavy metal accumulation between the sexes were also negligible. These results suggest that age-related vulnerability and environmental exposure may play a more substantial role in olfactory dysfunction than biological sex alone.

Rhinomanometric assessments of 97 patients in our study revealed only minimal differences in nasal airflow resistance between the group with olfactory dysfunction diagnosed prior to surgery (*n* = 55) and the control group without olfactory dysfunction (*n* = 42), although differences in olfactory function between the groups were present. The observed associations suggest that heavy metal accumulation in nasal mucosa may contribute to chronic inflammatory remodeling and olfactory dysfunction independently of mechanical nasal obstruction. The modest contribution of nasal airflow resistance to olfactory outcomes measured by rhinomanometry suggests the importance of sensorineural and tissue-level pathological mechanisms [36,37,38]. Therefore, in patients with chronic rhinosinusitis, assessment of environmental exposure history can provide clinically important information for the evaluation of olfactory dysfunction.

Previous occupational and environmental studies have documented olfactory deficits following exposure to airborne pollutants and heavy metals [12,17,19,20,21,22,23,24,39,40]. Most of these studies primarily focused on systemic neurotoxicity or lower respiratory tract sequelae [29,30,31], but direct quantitative assessment of heavy metal concentrations within human nasal mucosal tissue represents a critical gap in the research literature.

Consequently, the current study provides novel clinical evidence linking heavy metal deposition within nasal mucosal tissue to functional olfactory impairment, obtained by combining ICP–MS analysis with objective olfactometry, rhinomanometry, and histopathological assessment. Importantly, such integrated toxicological, functional, and histopathological nasal mucosal tissue analyses remain relatively scarce, and there are currently no standardized clinical monitoring approaches for patients with suspected environmentally associated olfactory dysfunction [8,10,17,19].

Among the analyzed metals—Cd, As, Pb, Cr, Mn, Ni, Cu, Zn, and Fe—in the nasal mucosa and secretions of patients diagnosed with nasal septal deviation, nasal polyposis, or chronic rhinosinusitis, Fe was identified as the strongest independent predictor of olfactory dysfunction, and its increased accumulation in chronic rhinosinusitis may have greater pathophysiological relevance. Despite its role as an essential micronutrient important for proper cellular metabolism, excessive local accumulation of Fe may promote oxidative stress, epithelial injury, and altered neuronal signaling through reactive oxygen species. Similarly, exposure to Pb and Cr and their accumulation in biological tissues may promote mitochondrial dysfunction, neuronal degeneration, chronic mucosal inflammation, and impaired olfactory neurotransmission [15,16,18,28,35]. Furthermore, statistical correlation analyses demonstrated positive associations among Cr, Mn, and Ni concentrations, indicating shared environmental or occupational exposure pathways that may contribute to these pathological processes.

The histopathological observations of our study substantiate the biological significance of heavy metal deposition within sinonasal tissue. MANOVA statistical analysis revealed a highly significant overall association between heavy metal profiles and pathohistological categories (Wilks’ Lambda = 0.038, F(36,316.52) = 12.177, *p* < 0.001), demonstrating that elemental accumulation patterns varied distinctively across tissue categories. Malignant tissue specimens (carcinoma) showed highly elevated Cr levels, whereas premalignant lesions showed substantially increased Cd levels. These findings demonstrate that the heavy metal distributions differed significantly across pathohistological categories of nasal mucosal tissue and pathohistological changes, although the study population was relatively small. Furthermore, these findings support previous experimental and epidemiological research studies identifying Cr and Cd as potent carcinogens capable of inducing oxidative stress, genotoxicity, epithelial dysplasia, and oncogenesis [15,16,18,28]. Notably, healthy respiratory mucosa was characterized by significantly elevated Fe, Cu, and Zn concentrations compared to dysplastic, metaplastic, and premalignant lesions. These findings underscore altered micronutrient metabolism, chronic inflammatory tissue changes, or compensatory protective epithelial responses in affected mucosal tissue, as confirmed in previous studies of chronic inhalational exposure to pollutants inducing pathohistological changes [27,28].

When the pathohistological findings were interpreted in the context of the corresponding clinical diagnoses, distinct heavy metal patterns emerged across the three clinical groups. Septal deviation, representing non-inflammatory mucosa, was characterized by the highest Fe and Cu concentrations, whereas CRSsNP demonstrated depletion of these essential trace elements during chronic inflammatory remodeling. In contrast, CRSwNP exhibited the most pronounced dysregulation, including elevated Cr, Ni, and Cd concentrations in tissues with advanced epithelial remodeling. Although this clinicopathological interpretation should be considered exploratory, it supports the concept that heavy metal accumulation may accompany progression from chronic inflammation to epithelial dysplasia.

The findings of this study further support the hypothesis that heavy metal accumulation participates in these pathological processes. The observed associations are supported by numerous biological mechanisms. Experimental studies demonstrated neurotoxic effects of inhaled nanoparticles and metal-containing aerosols via axonal transport from the olfactory mucosa to the olfactory bulb and central nervous system [13,14,41]. Chronic exposure subsequently leads to oxidative stress, epithelial barrier dysfunction, and impaired mucociliary clearance, resulting in inflammatory cytokine activation, neuronal degeneration, and altered olfactory neurotransmission in susceptible individuals [12,19,35]. This pathway links environmental exposure to the toxic effects of heavy metals, from sinonasal injury to broader neurological consequences [7,41,42].

Several limitations should be acknowledged. First, the number of carcinoma and precancerous lesions was relatively small, limiting the statistical power of subgroup analyses. Second, heavy metal concentrations were determined in surgically obtained tissue specimens at a single time point and therefore do not reflect cumulative lifetime exposure. Finally, although significant associations were observed, the observational design does not permit conclusions regarding causality.

## 5. Conclusions

This study demonstrated significant associations between heavy metal concentrations in the nasal mucosa and impaired olfactory function in 97 patients undergoing sinonasal surgery. Increased concentrations of Cr, Fe, and Pb were associated with lower preoperative olfactory scores, with Fe identified as the strongest independent predictor of olfactory dysfunction. Distinct heavy metal distribution patterns were observed across pathohistological tissue categories, with Cr concentrations highest in carcinoma and Cd concentrations markedly elevated in precancerous lesions. Older age was associated with poorer olfactory performance and strengthened the negative associations between selected heavy metals and olfactory function. These findings support the hypothesis that chronic environmental or occupational exposure to heavy metals may contribute to neuroepithelial injury and olfactory dysfunction in chronic rhinosinusitis. Further longitudinal studies incorporating detailed lifetime environmental, occupational, and residential exposure data are warranted to clarify causal mechanisms and to evaluate the clinical value of heavy metal monitoring in chronic rhinosinusitis and other sinonasal disorders.

## Figures and Tables

**Table 1:** Demographic and clinical characteristics of patients.

Variable	Total (*n* = 97)	Patients with Olfactory Dysfunction (*n* = 55)	Patients Without Olfactory Dysfunction (*n* = 42)	*p*-Value
Male, *n* (%)	58 (59.8)	34 (61.8)	24 (57.1)	0.64
Female, *n* (%)	39 (40.2)	21 (38.2)	18 (42.9)	
Age (years), mean ± SD	47.8 ± 15.1	49.6 ± 14.8	45.4 ± 15.2	0.18
Age 18–37 years, *n* (%)	32 (33.0)	15 (27.3)	17 (40.5)	
Age 38–55 years, *n* (%)	33 (34.0)	19 (34.5)	14 (33.3)	0.41
Age 56–78 years, *n* (%)	32 (33.0)	21 (38.2)	11 (26.2)	
Clinical diagnosis, *n* (%)				0.031
Septal deviation	42 (43.3)	20 (36.4)	25 (59.5)	
CRSsNP	28 (28.9)	18 (32.7)	10 (23.8)	
CRSwNP	24 (24.7)	17 (30.9)	7 (16.7)	
OL_BE	3.70 ± 1.27	3.40 ± 1.30	4.10 ± 1.20	<0.001
OL_AFT	4.95 ± 1.45	4.60 ± 1.30	5.10 ± 1.50	0.021
R1	0.52 ± 0.11	0.52 ± 0.10	0.53 ± 0.11	0.62
R2	0.39 ± 0.10	0.38 ± 0.10	0.40 ± 0.11	0.34

**Table 2:** Heavy metal concentrations in nasal mucosa samples (μg/g).

Metal	Males (*n* = 58)	Females (*n* = 39)	Age 18–37 (*n* = 32)	Age 38–55 (*n* = 33)	Age 56–78 (*n* = 32)	Patients with Olfactory Dysfunction (*n* = 55)	Patients Without Olfactory Dysfunction (*n* = 42)
Chromium (Cr)	0.22 ± 0.30	0.20 ± 0.28	0.18 ± 0.25	0.21 ± 0.29	0.25 ± 0.33	0.24 ± 0.31	0.21 ± 0.29
Iron (Fe)	110.0 ± 105.0	108.0 ± 100.0	115.0 ± 110.0	109.0 ± 105.0	107.0 ± 102.0	111.0 ± 107.0	106.0 ± 103.0
Cadmium (Cd)	0.02 ± 0.03	0.03 ± 0.05	0.02 ± 0.03	0.03 ± 0.05	0.03 ± 0.04	0.03 ± 0.04	0.03 ± 0.05
Nickel (Ni)	0.76 ± 2.25	0.68 ± 1.98	0.72 ± 2.10	0.71 ± 2.05	0.70 ± 2.00	0.74 ± 2.20	0.69 ± 1.95
Lead (Pb)	0.07 ± 0.15	0.08 ± 0.17	0.07 ± 0.16	0.08 ± 0.17	0.08 ± 0.16	0.07 ± 0.16	0.08 ± 0.17

**Table 3:** Heavy metal concentrations according to pathohistological findings.

Heavy Metal (μg/g)	Healthy Respiratory Mucosa (*n* = 42)	Metaplasia (*n* = 26)	Dysplasia (*n* = 22)	Precancerous Lesion (*n* = 5)	Carcinoma (*n* = 2)	*p*-Value
Cr	0.37 ± 0.58	0.18 ± 0.12	0.39 ± 0.71	0.06 ± 0.03	3.02 ± 2.08	<0.001
Mn	0.42 ± 0.41	0.38 ± 0.33	0.46 ± 0.61	0.55 ± 0.44	0.58 ± 0.52	0.284
Fe	278.4 ± 132.6	86.4 ± 59.5	120.5 ± 78.2	39.1 ± 18.5	266.7 ± 18.5	<0.001
As	0.018 ± 0.014	0.016 ± 0.011	0.021 ± 0.014	0.015 ± 0.010	0.031 ± 0.010	0.087
Cd	0.104 ± 0.062	0.028 ± 0.021	0.032 ± 0.027	17.03 ± 37.94	0.073 ± 0.024	<0.001
Ni	0.89 ± 1.02	0.82 ± 0.75	1.17 ± 2.61	0.97 ± 0.53	17.73 ± 9.84	0.062
Cu	2.30 ± 1.01	0.87 ± 0.42	1.19 ± 0.55	0.73 ± 0.29	2.48 ± 0.23	<0.001
Zn	8.74 ± 3.81	3.87 ± 2.24	5.22 ± 2.68	2.30 ± 1.29	5.36 ± 2.23	0.011
Pb	0.11 ± 0.15	0.05 ± 0.06	0.08 ± 0.09	0.03 ± 0.02	0.25 ± 0.29	0.174

**Table 4:** Significant Tukey HSD post hoc comparisons of heavy metal concentrations according to pathohistological findings.

Heavy Metal	Comparison Between Tissue Categories (Pathological and Non-Pathological)	Mean Difference (μg/g)	*p*-Value
Cr	Carcinoma vs. dysplasia	2.627	<0.001
Cr	Carcinoma vs. metaplasia	2.841	<0.001
Cr	Carcinoma vs. precancerous lesion	2.652	<0.001
Cr	Carcinoma vs. healthy respiratory mucosa	2.956	<0.001
Fe	Healthy respiratory mucosa vs. dysplasia	157.94	0.0016
Fe	Healthy respiratory mucosa vs. metaplasia	191.99	<0.001
Fe	Healthy respiratory mucosa vs. precancerous lesion	239.36	0.0004
Cd	Precancerous lesion vs. dysplasia	16.995	0.0017
Cd	Precancerous lesion vs. metaplasia	16.999	0.0011
Cd	Precancerous lesion vs. healthy respiratory mucosa	16.923	0.0146
Cu	Healthy respiratory epithelium vs. dysplasia	1.109	0.0108
Cu	Healthy respiratory epithelium vs. metaplasia	1.427	0.0003
Cu	Healthy respiratory epithelium vs. precancerous lesion	1.569	0.0071
Zn	Healthy respiratory epithelium vs. metaplasia	4.869	0.0250

**Table 5:** Heavy metal distribution according to clinical diagnosis and corresponding pathohistological findings.

Heavy Metal (μg/g)	Septal Deviation (Healthy Mucosa Phenotype)	CRSsNP (Metaplasia/Low-Grade Dysplasia)	CRSwNP (Dysplasia/Precancerous Lesion/Carcinoma Spectrum)
Cr	0.37 ± 0.58	0.18 ± 0.12	3.02 ± 2.08
Mn	0.42 ± 0.41	0.38 ± 0.33	0.55 ± 0.44
Fe	278.4 ± 132.6	86.4 ± 59.5	266.7 ± 18.5
As	0.018 ± 0.014	0.016 ± 0.011	0.031 ± 0.010
Cd	0.104 ± 0.062	0.028 ± 0.021	17.03 ± 37.94
Ni	0.89 ± 1.02	0.82 ± 0.75	17.73 ± 9.84
Cu	2.30 ± 1.01	0.87 ± 0.42	2.48 ± 0.23
Zn	8.74 ± 3.81	3.87 ± 2.24	5.36 ± 2.23
Pb	0.11 ± 0.15	0.05 ± 0.06	0.25 ± 0.29

Overall, septal deviation was characterized by preserved physiological concentrations of Fe, Cu, and Zn; CRSsNP by depletion of essential trace elements; and CRSwNP by progressive accumulation of Cr, Ni, and Cd, reflecting increasing epithelial remodeling and potential metal-associated tissue injury.

**Table 6:** Predictors of preoperative olfactory function (OL_BE).

**A. Heavy metals and demographic predictors**				
Predictor	β	SE	*p*	95% CI
Chromium (Cr)	−0.12	0.05	0.018 *	−0.22 to −0.02
Iron (Fe)	−0.08	0.03	0.009 *	−0.14 to −0.02
Lead (Pb)	−0.10	0.05	0.048 *	−0.20 to −0.00
Age (terciles)	−0.15	0.06	0.014 *	−0.27 to −0.03
Group (tested)	−0.20	0.07	0.005 *	−0.34 to −0.06
Gender (male)	0.05	0.04	0.21	−0.03 to 0.13
**B. Rhinomanometry predictors**				
Predictor	β	SE	*p*	95% CI
R1	0.45	0.12	<0.001 *	0.21 to 0.69
R2	0.30	0.10	0.003 *	0.10 to 0.50
Age	−0.10	0.05	0.048 *	−0.20 to −0.00
Group	−0.15	0.06	0.014 *	−0.27 to −0.03
Gender	0.05	0.04	0.21	−0.03 to 0.13
**C. Stepwise regression model**				
Step	Variable Entered	R^2^	ΔR^2^	*p*
1	Iron (Fe)	0.145	0.145	<0.001 *
2	Lead (Pb)	0.218	0.073	<0.001 *
3	Chromium (Cr)	0.268	0.050	<0.001 *
4	Age (terciles)	0.315	0.047	<0.001 *
**D. Interaction effects**				
Interaction Term	β	SE	*p*	
Fe × Age	−0.06	0.03	0.048 *	
Pb × Age	−0.08	0.04	0.048 *	
Cr × Age	−0.05	0.03	0.098	
Fe × Gender	−0.04	0.03	0.19	
Pb × Gender	−0.03	0.04	0.45	
Fe × Group	−0.07	0.04	0.083	
Pb × Group	−0.09	0.05	0.074	

* Significant at *p* < 0.05.

## Data Availability

The original contributions presented in this study are included in the article. Further inquiries can be directed to the corresponding author(s).

## Abbreviations

The following abbreviations are used in this manuscript:

ANOVA	analysis of variance
As	arsenic
Cd	cadmium
CHC	Clinical Hospital Center Rijeka (hrv. Klinički bolnički centar Rijeka)
CRS	chronic rhinosinusitis
CRSsNP	chronic rhinosinusitis with nasal polyps
CRSwNP	chronic rhinosinusitis without nasal polyps
Cr	chromium
Cu	copper
Fe	iron
FESS	functional endoscopic sinus surgery
Hg	mercury
HSD	honestly significant difference (Tukey’s test)
ICP-MS	inductively coupled plasma mass spectrometry
MANOVA	multivariate analysis of variance
Mn	manganese
Ni	nickel
OL_AFT	post-test olfactometry scores
OL_BE	baseline olfactory performance
Pb	lead
R1	rhinomanometry 1 (baseline measurement)
R2	rhinomanometry 2
SD SDOT	standard deviation Smell Diskettes Olfaction Test
TIPH PGKC	Teaching Institute of Public Health of Primorsko-Gorski Kotar County (*hrv. Nastavni zavod za javno zdravstvo Primorsko-goranske županije*)
Zn	zinc

## Appendix A

**Table A1:** Descriptive statistics of heavy metal concentrations by gender, age terciles, and group (μg/g).

Metal	Males (*n* = 58)	Females (*n* = 39)	Age 18–37 (*n* = 32)	Age 38–55 (*n* = 33)	Age 56–78 (*n* = 32)	Patients with Olfactory Dysfunction (*n* = 55)	Patients Without Olfactory Dysfunction (*n* = 42)
Chromium (Cr)	0.22 ± 0.30	0.20 ± 0.28	0.18 ± 0.25	0.21 ± 0.29	0.25 ± 0.33	0.24 ± 0.31	0.21 ± 0.29
Manganese (Mn)	0.40 ± 0.60	0.35 ± 0.55	0.38 ± 0.58	0.39 ± 0.59	0.42 ± 0.62	0.41 ± 0.60	0.36 ± 0.58
Iron (Fe)	110.0 ± 105.0	108.0 ± 100.0	115.0 ± 110.0	109.0 ± 105.0	107.0 ± 102.0	111.0 ± 107.0	106.0 ± 103.0
Arsenic (As)	0.02 ± 0.03	0.02 ± 0.03	0.02 ± 0.03	0.02 ± 0.03	0.02 ± 0.03	0.02 ± 0.03	0.02 ± 0.03
Cadmium (Cd)	0.02 ± 0.03	0.03 ± 0.05	0.02 ± 0.03	0.03 ± 0.05	0.03 ± 0.04	0.03 ± 0.04	0.03 ± 0.05
Nickel (Ni)	0.76 ± 2.25	0.68 ± 1.98	0.72 ± 2.10	0.71 ± 2.05	0.70 ± 2.00	0.74 ± 2.20	0.69 ± 1.95
Copper (Cu)	0.87 ± 0.90	1.10 ± 0.85	0.95 ± 0.88	0.98 ± 0.87	1.00 ± 0.86	0.92 ± 0.89	1.05 ± 0.84
Zinc (Zn)	4.50 ± 4.20	5.10 ± 4.50	4.70 ± 4.30	4.80 ± 4.35	4.90 ± 4.40	4.60 ± 4.25	5.00 ± 4.45
Lead (Pb)	0.07 ± 0.15	0.08 ± 0.17	0.07 ± 0.16	0.08 ± 0.17	0.08 ± 0.16	0.07 ± 0.16	0.08 ± 0.17

**Table A2:** Correlation matrix: heavy metals, rhinomanometry, and olfactometry.

Variable	Cr	Mn	Fe	As	Cd	Ni	Cu	Zn	Pb	R1	R2	OL_
Mn	0.42 *	—	—	—	—	—	—	—	—	—	—	—
Fe	0.38 *	0.51 *	—	—	—	—	—	—	—	—	—	—
As	0.15	0.22	0.18	—	—	—	—	—	—	—	—	—
Cd	0.25	0.31 *	0.28 *	0.42 *	—	—	—	—	—	—	—	—
Ni	0.19	0.26	0.21	0.38 *	0.55 *	—	—	—	—	—	—	—
Cu	0.32 *	0.38 *	0.35 *	0.29 *	0.41 *	0.48 *	—	—	—	—	—	—
Zn	0.28 *	0.35 *	0.32 *	0.25	0.38 *	0.44 *	0.62 *	—	—	—	—	—
Pb	0.41 *	0.48 *	0.45 *	0.32 *	0.52 *	0.51 *	0.58 *	0.55 *	—	—	—	—
R1	−0.18	−0.22	−0.25	−0.12	−0.15	−0.14	−0.20	−0.18	−0.28 *	—	—	—
R2	−0.15	−0.19	−0.22	−0.10	−0.12	−0.11	−0.17	−0.15	−0.24	0.68 *	—	—
OL_BE	−0.32 *	−0.28 *	−0.38 *	−0.15	−0.22	−0.18	−0.25	−0.21	−0.35 *	0.45 *	0.30 *	—

* *p* < 0.05.
